# Limited condition dependence of male acoustic signals in the grasshopper *Chorthippus biguttulus*

**DOI:** 10.1002/ece3.309

**Published:** 2012-07-11

**Authors:** Alexandra Franzke, Klaus Reinhold

**Affiliations:** Department of Evolutionary Biology, Bielefeld UniversityMorgenbreede 45, 33615, Bielefeld, Germany

**Keywords:** Acoustic signal traits, *Chorthippus biguttulus*, condition dependence, food plant quality

## Abstract

In many animal species, male acoustic signals serve to attract a mate and therefore often play a major role for male mating success. Male body condition is likely to be correlated with male acoustic signal traits, which signal male quality and provide choosy females indirect benefits. Environmental factors such as food quantity or quality can influence male body condition and therefore possibly lead to condition-dependent changes in the attractiveness of acoustic signals. Here, we test whether stressing food plants influences acoustic signal traits of males via condition-dependent expression of these traits. We examined four male song characteristics, which are vital for mate choice in females of the grasshopper *Chorthippus biguttulus*. Only one of the examined acoustic traits, loudness, was significantly altered by changing body condition because of drought- and moisture-related stress of food plants. No condition dependence could be observed for syllable to pause ratio, gap duration within syllables, and onset accentuation. We suggest that food plant stress and therefore food plant quality led to shifts in loudness of male grasshopper songs via body condition changes. The other three examined acoustic traits of males do not reflect male body condition induced by food plant quality.

## Introduction

According to the handicap hypothesis, exaggerated sexual traits should be preferred by females because these traits signal male genetic quality (Andersson 1994). In this context, a heightened condition-dependent expression of sexual ornaments is predicted (Pomiankowski 1987; Iwasa and Pomiankowski 1994), which could slow down the elimination of genetic variation. In line with this expectation, there is some evidence for condition dependence of sexual ornaments (Keyser and Hill 1999; David et al. 2000; Cotton et al. 2004). Further evidence exists that condition-dependent ornaments or displays contain reliable information about genotypic quality of the male (Rowe and Houle 1996; David et al. 2000). Females may therefore obtain indirect benefits from including condition-dependent male sexual signals in mate choice decisions (Andersson 1994; Johnstone 1995). Male body condition is likely to be strongly affected by environmental factors (Iwasa and Pomiankowski 1999; Hunt et al. 2004b). Therefore, male condition-dependent sexual ornaments are positively related to total fitness and thus can reliably indicate male quality (Hunt et al. 2004b). Males in good condition should be able to signal their quality through greater sexual trait size, whereas males in poor conditions should be unable to do this because of the viability costs associated with such costs.

Acoustic sexual signals represent sexual ornaments that play a major role in mammals (Waitt et al. 2003), birds (Thomas 2002), amphibians (Grafe 1996; Grafe and Thein 2001), and insects (Hoback and Wagner 1997; Reinhold 1999) for attracting mating partners (Searcy and Andersson 1986; Bradbury and Vehrencamp 1998). The production of acoustic signals is often very costly because increased metabolic energy is needed to produce calling songs, and often, honest signaling, for example, of immunocompetence leads to increased costs (Prestwich 1994; Hoback and Wagner 1997; Jacot et al. 2005; Munoz et al. 2010). For that reason, the likelihood of condition dependence increases (Stevens and Josephson 1977). In many orthopteran species, male songs serve to attract mating partners, and male song traits are an important predictor of male mating success (Heller and Helversen 1986; Brown et al. 1996; Holzer et al. 2003; Bentsen et al. 2006). Previous studies of acoustically communicating orthopterans found evidence for condition dependence in calling rate when using variation in food quantity or quality as treatment but no condition dependence for calling song characteristics (Wagner and Hoback 1999; Gray and Eckhardt 2001; Holzer et al. 2003; Hunt et al. 2004a). Our study should be a useful supplement embedded in a more ecological context in times of climate change. Moreover, we shed light on the question whether males get their tunes across in times of increasing urban noise if loudness is condition dependent.

In our study, we examined whether acoustic signal traits of the grasshopper species *Chorthippus biguttulus* might be condition dependent. We experimentally manipulated body condition of grasshoppers by varying food plant quality. Plant quality was manipulated by water stress of plants. Variation in diet quality is known to play an important role for male body condition and female reproductive success in *C. biguttulus* (Franzke and Reinhold 2011). In this study, we observed that plants growing under drought stress conditions contained higher nutrient contents than plants growing under moisture stress conditions. Grasshoppers that fed on high-quality plants (drought stress) performed better than grasshoppers reared on poor-quality plants (moisture stress). Thus, it is quite possible, also in accordance with the studies listed above, that plant quality can influence acoustic signal traits which are important for female mate choice via body condition. In line with these arguments, we examined the hypotheses that grasshopper acoustic signal traits are expressed in a condition-dependent manner via plant quality changes.

## Material and Methods

### Study organism

**Figure 1:**
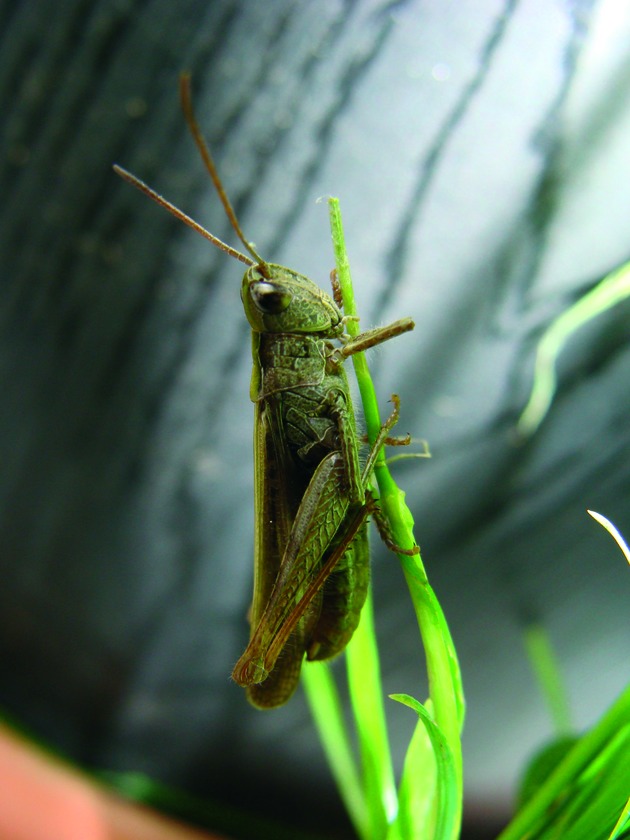
*Chorthippus biguttulus*.

*Chorthippus biguttulus* (Acrididae, Gomphocerinae; Linnaeus 1758; Fig. 1) is an acoustically communicating gomphocerine grasshopper in which pair formation is achieved by duetting between the sexes. Females use song characteristics for species recognition and mate choice (von Helversen and von Helversen 1994; Safi et al. 2006). Male songs usually consist of several verses, each having a length of about 2–3 sec. Each verse has a distinctive syllable–pause structure, with syllable duration ranging from about 40 to 100 msec and pause duration from 8 to 25 msec, depending on the individual and on temperature during stridulation (von Helversen 1972; von Helversen and von Helversen 1997). Females use a number of features for song evaluation. These include (1) the presence of low- (6–8 kHz) and high- (20–30 kHz) frequency components in the song spectrum, (2) a minimum syllable duration of about 40 msec, (3) an optimum syllable–pause duration ratio of about 5:1, (4) gap duration within syllables less than 2 msec, (5) preferably high song loudness, and (6) preferably high onset accentuations (von Helversen 1972; Helversen and Helversen 1997; Klappert and Reinhold 2003; von Helversen et al. 2004).

We used individuals from two populations of *C. biguttulus*, one of the most abundant grasshopper species in Central Europe (Ingrisch and Köhler 1998; Maas et al. 2002). We caught 40 adult females in each of the two populations in August 2007 in the Northwest of Germany, near the city of Bielefeld. The first population was collected on a bank of the highway A33 in the direction “Osnabrück” near “Erpen,” “Dissen am Teutoburger Wald” (52°07′23′′N, 8°10′42′′E) and the second population in about 2 km distance from the first population close to the highway A33 near “Erpen,” “Dissen am Teutoburger Wald” (52°07′33′′N, 8°09′50′′E). Females were kept separately in plastic terraria (18 × 11 × 13 cm) for 3 weeks in the laboratory. A field-cut grass mixture served as food. For oviposition, each terrarium contained small plastic cups filled with a mixture of moist sand and soil (1:1). Four weeks after the last oviposition, the plastic cups with egg cases were transferred to the climatic exposure test cabinet where they were stored at 4°C until the start of the experiment in May 2008.

### Experimental setup

Equal mixtures of five food plants (*Agrostis capillaris*, *Dactylis glomerata*, *Festuca rubra*, *Poa pratensis*, *Trifolium pratense*) were applied for three plant treatments to test for the effects of different plant water availabilities on grasshoppers in a greenhouse. Different volumes of water were provided to plant treatments. The drought stress treatment represented about 60% less precipitation than the control which represented an average German summer precipitation of 239 L/m² according to the German Meteorological Service, and the moisture stress treatment was saturated once every day (about 40% more precipitation than the control). For all plant treatments, water was applied daily at ground level in different quantities depending on the plant water treatment.

Plants were sown in plastic containers (60 × 40 × 15 cm) in mid-February 2008 containing a soil mixture of vermiculite and “Wesersand” from the river “Weser” in Germany (1:1). Plant treatments consisted of 15 containers each (five containers as reserve). Until the start of the experiment, plant containers were watered as required and once a week with 1 L of a 50% concentration of a modified Hoagland nutrient solution for plants (Hoagland and Amon 1950). One week before starting the experiment, plant containers were randomly separated into the three experimental treatment groups with their allocated water regimes.

To allow hatching of the nymphs, egg cases were moved into a climatic chamber with a temperature of 26°C, at the beginning of May 2008. After hatching of grasshopper nymphs (14 or 15 days), all of them were directly put in wooden cages (40 × 27 × 32 cm), placed above the plant containers. Hence, about 300 grasshopper nymphs, males and females, of each of the two populations were randomly assigned to the three plant water treatments, so that about 100 grasshoppers of each population were distributed into each treatment (20 grasshopper nymphs per cage, meaning 5 cages per treatment and population and altogether 10 cages per treatment, meaning 30 cages in total). Five plant containers were added to the experiment as reserve. Once in a week, containers were rotated and transferred to another place, cages were put on another plant container (also at reserve containers), and each group of grasshoppers was transferred into another cage. The procedure was used to avoid position effects and effects of microclimatic variation. Grasshoppers could feed on the same type of plant treatment from hatching until 3 weeks after the final molt. For each grasshopper, we calculated developmental time as the time from hatching until the final molt. As an estimation of body size, we measured the left femur length of adult grasshoppers with an ocular micrometer under a stereomicroscope and recorded males' weight 1 day after the final molt.

Temperature and humidity in the cages were measured and recorded over the whole time of the experiment. Both parameters differed between treatments only in the night in the air layer near the ground, but at night, grasshoppers usually were on the ceiling of the cages. We therefore assume that direct effects of temperature and humidity had negligible effects on grasshoppers and were under controlled conditions.

### Male songs

Male songs were recorded on an IBM (Armonk, New York, USA) ThinkPad R51 laptop with Avisoft Bioacoustics 4.3 (Berlin, Germany) with a sampling rate of 192 kHz and a loudness encoding of 8 bits. The recording time of male individuals was 12–17 days after the imaginal molt. Males of the three treatment groups were recorded in a randomized order. We did not record one-legged males. All recorded songs were saved as WAV audio files. Songs of 177 individuals (drought = 67, control = 53, moisture = 57) were recorded in a climate chamber with a temperature of 28°C and a humidity of 40%. Individuals were placed in a sound insulated box on a frame of gauze positioned on a plastic cup filled with sand. A spotlight was adjusted above the grasshopper to light and warm the central position, which gave the animal an incentive to stay there. On the right side, lateral (90° angle horizontally) to the individual, a microphone (Bruel & Kjaer Type 4939, ¼ Microphone Free Field with a NC*MX-HD cable connector by Neutrik, Bremen, Germany) was positioned at a distance of 10 cm to the grasshopper. The distance from the microphone and position of the male were standardized so that the amplitude could be recorded accurately. To stimulate males for singing, a female grasshopper was placed next to the male. For each male, a female of the same treatment was randomly chosen. Five songs were recorded with two to five verses of each male. After recording, various acoustic signal traits were analyzed using a custom-built graphical user interface (GUI), based on MATLAB 2008a. We calibrated the recording system using the microphone of Bruel & Kjaer and a reference signal of 86.2 dB (SPL, sound pressure level) and a frequency of 10 kHz. In order to obtain the reference signal, we picked the loudest male song recorded for the present data set and recorded the same signal again in a distance of 10 cm to the loud speaker (Ultrasonic Dynamic Speaker ScanSpeak by Avisoft Bioacoustics) with a sound level meter (PeakTech 5035, 4 in 1 Multifunction environment tester by Conrad Electronic GmbH, Hirschau, Germany). All other signals were then referenced to the signal of the loudest male.

### Song analyses

**Figure 2:**
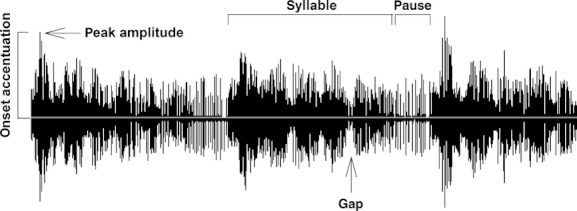
Oscillogram of a male chirp with the measured acoustic signal traits.

We analyzed four different acoustic signal traits (Fig. 2) which have previously been reported to determine song attractiveness for females as explained in section one of the material and methods: (1) the peak amplitude of the syllables (loudness), (2) the syllable to pause duration ratio, (3) the gap duration within syllables (syllables are not continuous but interrupted by short gaps), and (4) the onset accentuation of the syllables. We estimated all parameters by extracting the root mean square amplitude envelope of a phrase with an integration time of a third of a millisecond. The syllable onset accentuation was calculated by dividing the maximum syllable amplitude by the average amplitude of a syllable and transforming this ratio to a dB value by using the log_10_ and multiplying it by 20. All parameters are represented in Figure 2.

### Statistical analyses

To analyze the effect of treatments on all used grasshopper call parameters in this study, linear mixed models (LME) were performed using R version 2.15.0 with “body condition” and “developmental time” as covariates and “treatment” (a factor with the three levels: drought, control, and moisture) as fixed effects. As random effects, we used “male song” nested in “individual” nested in “group of grasshoppers” nested in “population.” We used the R packages “nlme” and “lme4” with the function “lme.” Interactions were tested but only remained in the model in case they were found to be significant. Likelihood ratio tests were run for significance testing. All dependent data were tested for normality and heteroscedasticity with the Lilliefors test and log transformed, if necessary. Body condition is given as a ratio between body weight and femur length of grasshoppers. As a correction for multiple testing did not change significance for the parameters, we do not give Bonferroni corrected *P*-values. Means are always displayed with standard errors (SE).

## Results

**Figure 3:**
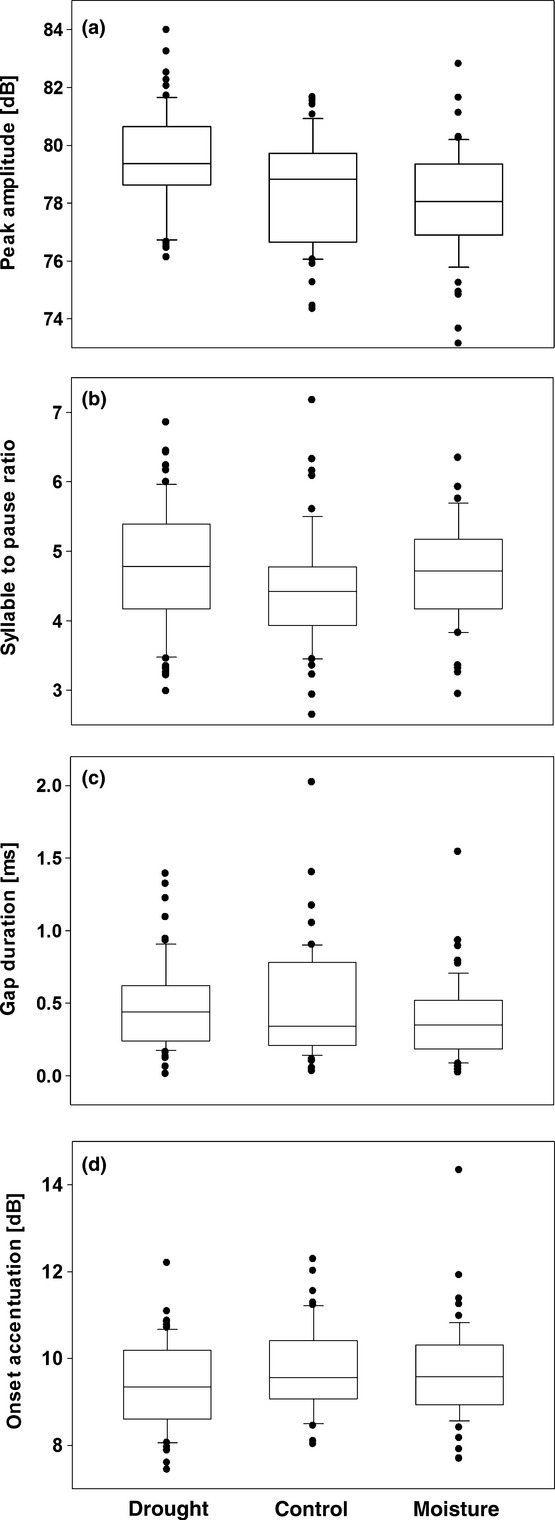
Acoustic signal traits as a function of the food plant water treatment: (a) peak amplitude of syllables, (b) syllable to pause ratio, (c) gap duration, (d) onset accentuation. Sample size: drought = 67, control = 53, and moisture = 57. Black bars represent medians, boxes quartiles, whiskers represent maximum and minimum, or 1.5 times the interquartile range in case of outliners.

The peak amplitude of syllables (loudness) differed significantly between food plant treatments (LME: *F*_2,26_ = 10.70, *P* < 0.001, Fig. 3a) and depended significantly on body condition and developmental time (LME: body condition, *F*_1,140_ = 4.39, *P* = 0.038; developmental time, *F*_1,146_ = 20.70, *P* < 0.001; Fig. 3). Males of the drought stress treatment were singing on average 1.45 dB louder than males of the moisture stress treatment (Table 1). The syllable to pause ratio, gap duration, and onset accentuation differed not significantly between males of the different treatments (LME: syllable to pause ratio, *F*_2,26_ = 2.08, *P* = 0.146; gap duration, *F*_2,26_ = 2.42, *P* = 0.109; onset accentuation, *F*_2,26_ = 2.52, *P* = 0.100; Fig. 3b–d) and were not condition dependent. Mean values of all listed parameters are shown in Table 1.

**Table 1:** Means of body size, developmental time, and acoustic signal traits of *Chorthippus biguttulus* grasshoppers of the three plant water treatments (drought, control, moisture)

Variable	Drought Mean ± SE (*N*)	Control Mean ± SE (*N*)	Moisture Mean ± SE (*N*)
Condition (mg/mm²)	0.938 ± 0.01 (64)	0.938 ± 0.01 (53)	0.951 ± 0.01 (54)
Developmental time (days)	39.60 ± 0.28 (67)	43.15 ± 0.30 (53)	44.26 ± 0.32 (57)
Peak amplitude (dB)	79.49 ± 0.21 (67)	78.42 ± 0.26 (53)	78.04 ± 0.25 (57)
Syllable to pause ratio	4.80 ± 0.12 (67)	4.43 ± 0.12 (53)	4.74 ± 0.12 (57)
Gap duration (msec)	0.49 ± 0.04 (67)	0.49 ± 0.05 (53)	0.38 ± 0.04 (57)
Onset accentuation (dB)	9.35 ± 0.12 (67)	9.74 ± 0.15 (53)	9.68 ± 0.14 (57)

Populations were pooled. *N*, number of males.

## Discussion

**Figure 4:**
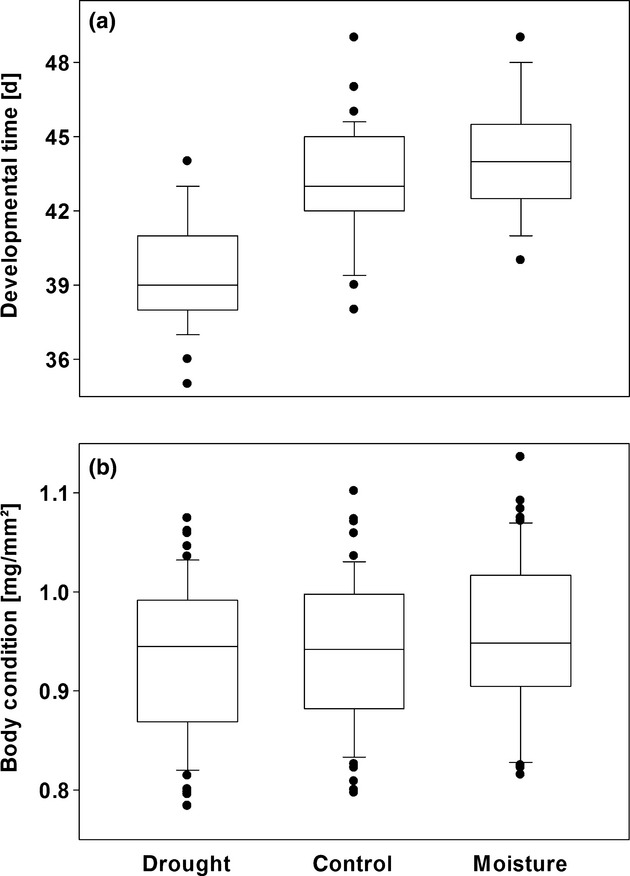
Life-history traits as a function of the food plant water treatment: (a) body condition, (b) developmental time. Sample size: body condition, drought = 64, control = 53, and moisture = 54; developmental time, drought = 67, control = 53, and moisture = 57. Black bars represent medians, boxes quartiles, whiskers represent maximum and minimum, or 1.5 times the interquartile range in case of outliners.

Plant quality due to varied water availability influenced male body condition and developmental time (Franzke and Reinhold 2011; Fig. 4, Table 1). These two parameters in turn had an influence on loudness, one of the four examined acoustic signal traits. The three other signal traits (syllable to pause ratio, gap duration, and onset accentuation) – all three important for sexual selection in *C. biguttulus* (Klappert and Reinhold 2003; von Helversen et al. 2004) – did not depend on body condition and developmental time. The observed influence of developmental time on loudness can be explained because developmental time is likely to be influenced by the treatment via its association with body size and itself has an effect on loudness.

The difference in loudness between males of the different treatments was limited to less than 1.5 dB. That an apparently small difference in dB value might be resolved by females seems implausible. However, in a previous study by von Helversen (1984) with *C. biguttulus*, it could be recognized, in a distance of 1 m to the loudspeaker, that females' response was saturated at about 6–8 dB above the threshold intensity. A 1.5 dB increase in loudness resulted in about 20% more reaction (frequency of females' acoustic responses) above threshold intensity. To estimate the effect of signal amplitude on male attractiveness and reproductive success, we used the data from Klappert and Reinhold (2003). To receive the effect of loudness on attractiveness of males, we log transformed the *x*-axis of the data given in Figure 3a in Klappert and Reinhold (2003) to obtain dB values and calculated the expected effect of a change in amplitude by 1.45 dB on attractiveness. The obtained average effects of plant quality on signal amplitude could accordingly be estimated to result in an increase in male fitness of about 9.5%, when drought conditions are compared with moisture conditions. As loudness is important in determining male mating success – exaggerated loudness will increase the probability to be detected by females and thereby the probability to mate – increased loudness should therefore lead to higher male mating success (Galliart and Shaw 1991; Klappert and Reinhold 2003).

Syllable to pause ratio, gap duration, and onset accentuation were not condition dependent. It might be assumed that this fact does not reflect heritable variation in fitness, as it is suggested for secondary sexual traits by a model of Rowe and Houle (1996) and has experimentally been shown for ornament expression in stalk-eyed flies (David et al. 2000) and dung beetles (Kotiaho et al. 2001). Compared with the effects of the treatments on female fitness (Franzke and Reinhold 2011) – female fitness under plant moisture conditions was reduced by about 50% compared with plant drought conditions – for now it is quite surprising that the examined acoustic signal traits were at maximum only slightly affected by the treatment, although according to our hypothesis male signal traits were expected to reflect male body condition and quality. The three noncondition-dependent acoustic traits which signal attractiveness of *C. biguttulus* males seem to be buffered against body condition induced by environmental changes. This fixedness of signal traits might be a benefit in rapidly and extreme changing environments like under climate change, because the genetic quality is not easily modified and is likely to be favored in poor-quality environments. Apart from the three attractiveness traits that were unaffected by body condition, loudness of male songs was condition dependent and the probability for males to receive acoustic responses by females increases with louder singing. Besides, natural background sound or noisy habitats might be reducing the ability of a female to discriminate between male calls on the basis of signal traits which could be observed in birds, frogs, and insects (Gerhardt and Huber 2002; Wollerman and Wiley 2002; Hammond and Winston 2003; Brumm and Naguib 2009; Samarra et al. 2009), whereby loudness might be the first signal trait that females perceive from males.

To summarize, signal traits contributing to the attractiveness of *C. biguttulus*, males show limited condition dependence. Loudness in *C. biguttulus* males is condition dependent whereby louder singing might be beneficial especially in noisy habitats to find a mating partner. If urban noise environments would be coupled with negative climatic variations, males are likely to have difficulties to get their tunes across and might have problems to be perceived by females. Even if males would be singing louder, females would also choose males based on the three other acoustic traits which are condition independent. However, if these signal traits are buffered against condition changes, selection might deplete the additive genetic variation in these traits quickly. In natural rapidly changing environments, condition independence of sexual selected traits is likely to be beneficial especially when environments become worse for organisms. This would mean that regardless of whether the environment influences condition positively or negatively females might select males according to their genetic material; therefore, sexual selection would be buffered against environmental changes.
